# The prognostic value of secreted frizzled-related protein 2 in elderly patients with heart failure: a single-center retrospective study

**DOI:** 10.1038/s41598-025-11765-6

**Published:** 2025-08-04

**Authors:** Peng Yu, Kailing Lin, Liman Wang, Chaochao Deng, Wenfei Zeng, Huizhen Yu

**Affiliations:** 1 Department of Geriatric Medicine, Shengli Clinical Medical College of Fujian Medical University, Fuzhou University Affiliated Provincial Hospital, Fuzhou, 350001 Fujian China; 2 Department of Cardiology in Jinshan Branch, Shengli Clinical Medical College of Fujian Medical University, Fuzhou University Affiliated Provincial Hospital, Fujian Provincial Hospital, Fuzhou, 350001 Fujian China; 3 Shengli Clinical Medical College of Fujian Medical University, Fuzhou, 350001 Fujian China; 4 Department of Pharmacy, Fujian Provincial Hospital, Fuzhou, 350001 Fujian China

**Keywords:** Secreted frizzled-related protein 2, Heart failure, Prognosis, Elderly, Cardiology, Cardiovascular diseases, Congenital heart defects, Heart failure

## Abstract

This study was conducted to assess the prognostic value of secreted frizzled-related protein 2 (SFRP2) for mortality and readmission in elderly patients with acute exacerbation of chronic heart failure (HF). Elderly patients hospitalized for worsening chronic HF were enrolled in the present study. We detected the concentration of serum SFRP2 in these patients. The primary endpoint of this study was defined as all-cause mortality and the secondary endpoint was a composite of all-cause mortality and readmission due to HF, acute myocardial infarction, and malignant arrhythmia during a median follow-up period of 450 (interquartile range 224–942) days. Multivariable Cox proportional hazard models were performed to evaluate the prognostic value of SFRP2. Of 161 patients at baseline, we observed 72 events (25 deaths and 47 readmissions). Serum SFRP2 levels were significantly elevated in elderly HF patients with events relative to those without and control subjects (all *P* < 0.001). The Kaplan-Meier analysis showed a significantly increased risk of all-cause mortality and cardiovascular readmissions stratified by the optimal cut-off value of serum SFRP2 level (log-rank *P* < 0.005). An elevated SFRP2 level and N-terminal pro-B-type natriuretic peptide (NT-proBNP) level was independently and significantly associated with the primary endpoint and secondary endpoint (adjusted hazard ratio [HR] 2.334, 95% confidence interval [CI] 1.059–5.147;*P* = 0.036 and HR 2.326, 95% CI 1.426–3.794;*P* = 0.001, respectively) in multivariable Cox regression analysis. A higher level of serum SFRP2 can be considered as an independent predictor of poorer clinical outcomes for elderly patients with acute exacerbation of chronic heart failure, indicating that evaluation of SFRP2 could provide more useful information for the long-term prognosis in these patients beyond NT-proBNP.

## Introduction

Heart failure (HF) is markedly increasing worldwide, particularly in countries with an improved life expectancy and a rapidly aging population^1^. Furthermore, heart failure is the end stage of heart disease characterized by higher mortality and rehospitalization rates. At present, N-terminal pro B-type natriuretic peptide (NT-proBNP) is considered to be a robust predictor of adverse outcomes in patients with HF^2^. However, NT-proBNP is susceptible to age, renal dysfunction, obesity and should be cautiously interpreted in the elderly patients^2,3^.Therefore, it is clinically valuable to explore novel cardiac biomarkers for early identification of prognostic risk factors in elderly patients with HF.

Wnt signaling pathway have many critical roles in regulating the cellular processes during embryonic and disease development by the frizzled family of seven-pass transmembrane receptors. Secreted frizzled-related protein 2 (SFRP2) that structurally resembles the Wnt frizzled receptors is a promising biomarker and is known as a key mediator of the Wnt signaling^4–7^. SFRP2, a Wnt signaling antagonist, modulates myocardial fibrosis and inflammation by regulating β-catenin pathways. In decompensated HF, elevated SFRP2 levels correlate with extracellular matrix remodeling and impaired cardiac repair mechanisms^8^. Previous studies emphasize the importance of SFRP2 in myocardial fibrosis and abnormalities of cardiac function^9–11^. Nevertheless, there is only a limited amount of information on the association of SFRP2 in real-world patients with HF. Data on SFRP2 in patients with HF have been greatly inconsistent in two recent studies. Yang et al.. reported that the serum level of SFRP2 was higher in HF patients who experienced primary outcome events^12^. Another study demonstrated that higher serum SFRP2 was significantly associated to lower odds of HF in patients with cardiovascular diseases^13^. SFRP2 may participate in cardiac diseases through myocardial fibrosis and abnormalities of cardiac function mechanisms, but clinical data directly linking SFRP2 to HF remain limited.Therefore, this study was conducted to verify whether SFRP2 can provide additional prognostic information for heart failure patients, independent of NT-proBNP.

Most patients admitted to hospitals with HF are elderly. Older HF patients are more prone to worse outcomes.The performance of SFRP2 at admission in elderly patients with chronic HF has not been fully elucidated.As such, our study was to analyze serum SFRP2 levels in elderly patients with worsening chronic HF, and explore the ability of circulating SFRP2 to predict subsequent adverse events.

## Methods

### Study population

**Fig. 1:**
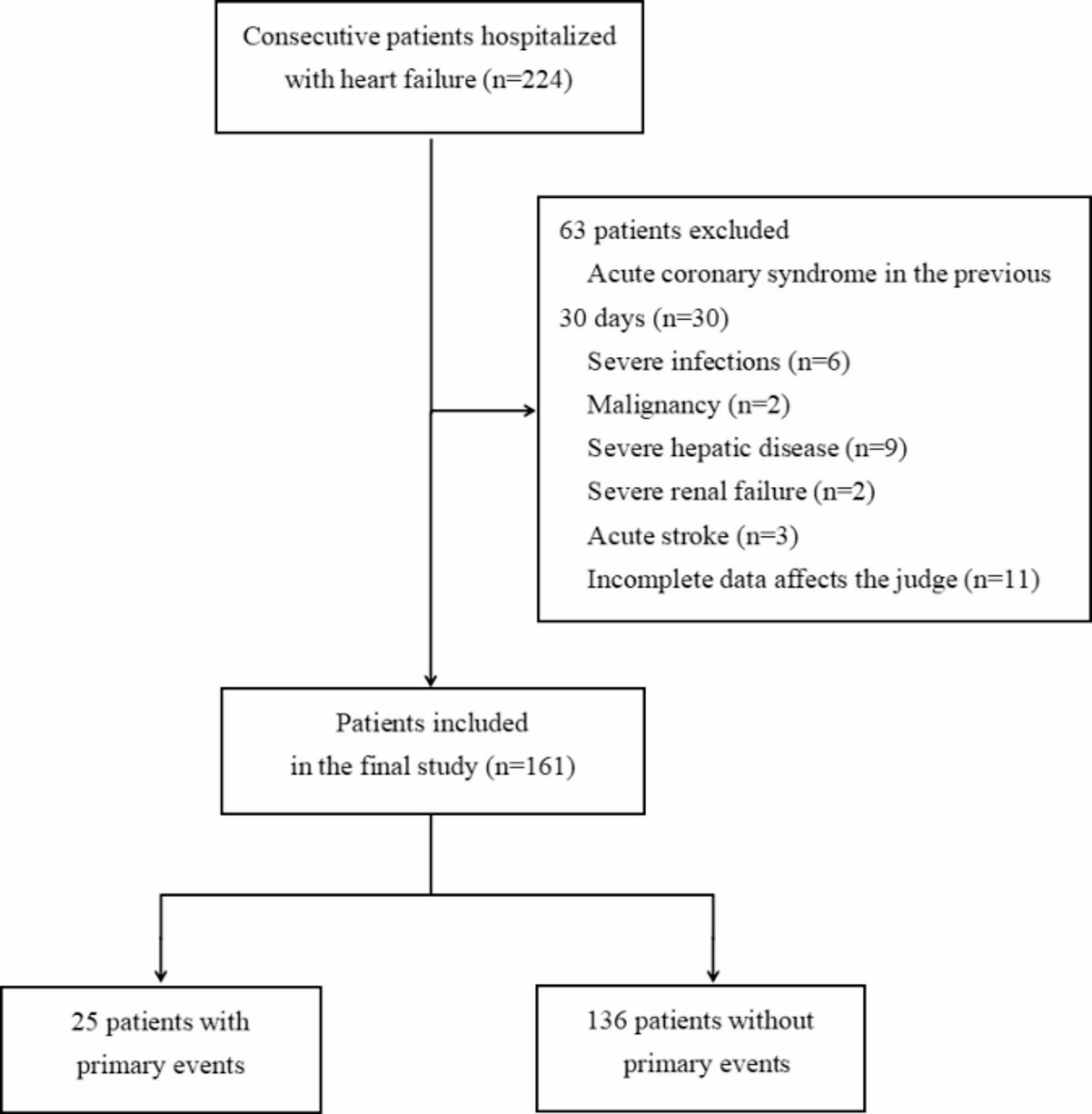
Flow diagram of the study.

Between January 2019 and October 2021, we initially enrolled 224 patients aged ≥ 60, who were consecutively admitted with acute exacerbation of chronic HF as diagnosed by the Framingham criteria^14^ to the cardiology department of Fujian Provincial Hospital (Fig. 1). Of these, 63 were excluded; 30 due to acute coronary syndrome in the previous 30 days, six for severe infections, two for a history of malignancy, nine for severe hepatic disease (bilirubin > 3× the upper limit of normal, or aspartate aminotransferase/alanine aminotransferase > 5× the upper limit of normal, or cirrhosis), two for severe renal failure (estimated glomerular filtration rate (eGFR) < 30mL/min/1.73m^2^ or under renal replacement therapy), three for acute stroke and eleven for the incomplete data affects the judge. Finally, a total of 161 patients with New York Heart Association (NYHA) functional class II-IV were studied. Patients were compared with 55 age-and gender-matched control subjects without history of cardiovascular disease from the healthcare center of Fujian Provincial Hospital. In the present study, all patients were treated in accordance with the principles recommended by the Chinese guidelines on HF^15^. The investigation conforms with the ethical guidelines outlined by the Declaration of Helsinki. All participants were provided informed consent during the study, which was approved by the hospital’s ethics committee (approval number: K2019-05-032).

### Baseline characteristics collection

We collected baseline characteristics including patient demographics, lifestyle habits, comorbidities, prescribed medications, vital signs, NYHA functional class as indicated by ESC guidelines^2^, HF etiology, biochemistry tests, and echocardiographic data from the hospital medical records. Body mass index (BMI) was calculated by the ratio between weight in kilograms and height in meters squared (kg/m^2^).

### Biomarker measurement

After at least 12 h of overnight fasting, the peripheral venous blood specimens of all subjects were obtained on the next day after admission and were used for laboratory tests immediately. At the same time, excess of serum sample were extracted and stored at -20℃ for up to 7 days and then sent to -80℃ refrigerator for long-term storage until required. Serum total SFRP2 levels were measured by an enzyme-linked immunosorbent assay (ELISA) kit, in accordance with the operating procedures of the kit instructions. The analytical range is 2.5–80.0 ng/mL. The intra-assay and inter-assay variations for ELISA were 10% and 15%, respectively. ELISA kit was purchased from Wuhan Ousaid Biological Company (No. QSD-H1038). Plasma concentration of NT-proBNP was determined with the Elecsys 2010 automated electrochemical immuno-analyzer (Roche Diagnostics K.K., Tokyo, Japan) and the experiment was performed by standard methods in the clinical laboratory of Fujian Provincial Hospital.

Complete blood counts (e.g., white blood cell and neutrophil) and hemoglobin level were all performed with a JMXN-3000 automated hematology analyzer (Sysmex Corporation, Kobe, Japan). Standard laboratory evaluations determined the serum levels of albumin (ALB), alanine aminotransferase (ALT), aspartate aminotransferase (AST), triglyceride (TG), total cholesterol (TC), high-density lipoprotein cholesterol (HDL-C), low-density lipoprotein cholesterol (LDL-C), serum creatinine, serum sodium (Na^+^), and serum potassium (K^+^) using a Jcobas 8000 autoanalyzer (Roche Diagnostics, Mannheim, Germany). Renal function was expressed as the eGFR which was calculated using the MDRD (Modification of Diet in Renal Disease equation adapted for Chinese patients) formula^16^.

### Echocardiographic data

Echocardiographic examination was usually performed by the experienced sonographer at a single center using the Vivid E9 echocardiography system (GE Healthcare, Milwaukee, WI, USA). Left ventricular ejection fraction (LVEF) was obtained from two-dimensional guided M-mode echocardiography and tested with a modified Simpson’smethod. Left atrial diameter (LAD), left ventricular diameter (LVD), right atrial diameter (RAD), and right ventricular diameter (RVD), as well as left ventricular end diastolic diameter (LVEDD) were also estimated. The above measurements were according to recommendations by the American and European Societies of Echocardiography.

### Follow-up and assessment of clinical outcomes

After discharge, follow-up of subjects was conducted through directing contact in an outpatient setting, structured telephone interview with the patients (or their family members if patients were deceased) or review of the available electronic health records. The follow-up duration was up to February 28, 2022. The primary endpoint of this study was defined as all-cause mortality including cardiac based death and non-cardiac death. The secondary endpoint was a composite of all-cause mortality and readmission due to HF, acute myocardial infarction, and malignant arrhythmia.

### Statistical analysis

All statistical analyses were completed using SPSS version 25.0 (IBM SPSS Inc., Chicago, USA) and Rversion 4.2.0, with the survminer package. Normality was confirmed using the Kolmogorov-Smirnov test in each group. Continuous variables were presented as mean ± standard deviation (SD) if normally distributed or median with 25th and 75th interquartile range (IQR)if skewed distributed. Categorical variables were exhibited as frequencies and percentages. When biomarkers had skewed distributions, they were log_10_-transformed(lg-transformed) to establish normality for further analyses. The baseline difference in characteristics between control subjects and patients with worsening chronic HF, or patients with or without events, were compared using the independent-sample Student’s *t-*test or Mann-Whitney *U*-test depending on the normality of the distribution. Categorical data were analyzed by Pearson’s chi-squared test or Fisher’s exact test.

Receiver operating characteristic (ROC) curve analysis was used to diagnose HF. The area under the curve (AUC) was computed together with 95% confidence interval (CI).A binomial logistic regression model was analysed to identify risk factors for HF, presented as odds ratio (OR) and 95% CI. The ROC analyses with AUCs were performed to identify precise cut-off values of SFRP2 and NT-proBNP that would best discriminate primary endpoint and the secondary endpoint in HF patients. Kaplan-Meier survival curves based on cut-off points determined according to the Youden index, were used to illustrate event-free and overall survival of patients with worsening chronic HF by log-rank test. Cox proportional hazard regression models were constructed to assess the independent clinical risk predictors of outcomes. The multicollinearity was assessed between covariates. If the variance inflation factor (VIF) ≤ 5, it indicated no severe multicollinearity issues. If the VIF>5, we would employ stepwise regression for variable selection. After making adjustments for confounding factors, hazard ratio (HR) with 95% CI was calculated. Those risk factors with a *P* < 0.05 in univariable analyses were entered into a forward stepwise multivariable Cox regression model. All *P* values were two-sided, and differences with *P* < 0.05 were considered to be statistically significant.

## Results

### Baseline characteristics of the studied population

In total, 161 patients with worsening chronic HF (74.89 ± 8.45 years, 59.00% male patients) and 55 normal controls of similar age and gender (74.98 ± 5.41 years, 50.90% male patients) were enrolled in the study. The baseline characteristics of patients with worsening chronic HF and control population were summarized in Table 1. There was no significant difference in SBP, DBP, BMI, and WBC between the two groups, but haemoglobin, median TC level, and eGFR (all *P* < 0.001) were significantly lower in the patients with worsening chronic HF than in controls. As expected, the median concentration of SFRP2 (22.97 ng/mL vs. 7.43 ng/mL, respectively) and NT-proBNP (3439.00 pg/mL vs. 64.43 pg/mL, respectively) were significantly higher in the HF group than those in the control group (both *P* < 0.001). Linear regression analysis showed no correlation between SFRP2 with age, creatinine levels and ejection fraction(*R* = 0.063, 0.071, 0.017, all *P >* 0.05).

**Table 1:** Baseline characteristics of HF patients and controls of matched age and gender.

	HF patients (*n* = 161)	Controls (*n* = 55)	Ρ value
Age (years)	74.89 ± 8.45	74.98 ± 5.41	0.930
Male (n,%)	95(59.00)	28 (50.90)	0.295
SBP (mmHg)	132.00 (119.50,148.50)	130.00 (117.00,141.00)	0.575
DBP (mmHg)	74.00 (66.00,85.50)	77.00 (71.00,88.00)	0.138
BMI (kg/m^2^)	23.31 (21.43,25.40)	23.67 (21.00,25.97)	0.611
WBC (×10^9^)	6.80 (5.40,8.50)	6.40 (5.80,7.50)	0.156
Hb (g/L)	124.44 ± 23.49	146.57 ± 14.00	< 0.001
TC (mmol/L)	3.87 (3.17,4.86)	5.54 (5.03,6.27)	< 0.001
eGFR (mL/min×1.73m^2^)	68.72 ± 29.46	90.35 ± 14.93	< 0.001
NT-proBNP(pg/mL)	3439.00(1755.50,5470.00)	64.43(33.80,106.00)	< 0.001
SFRP2 (ng/mL)	22.97 (14.26,33.13)	7.43 (4.24,13.24)	< 0.001

Continuous variables are reported as mean (standard deviation) or median (interquartile range). SBP, systolic blood pressure; DBP, diastolic blood pressure; BMI, body mass index; WBC, white blood cell; Hb, haemoglobin; TC, total cholesterol; eGFR, estimated glomerular filtration rate; SFRP2, secreted frizzled-related protein 2. P values are comparison between HF and control group.

### Diagnostic value of SFRP2 levels in elderly patient with worsening chronic HF

The diagnostic ability of SFRP2 to distinguish HF from non-HF was also analysed. By ROC *analysis*, SFRP2 had an AUC of 0.821 (95% CI 0.748–0.895,*P* < 0.001). The best cut-off value of SFRP2 for diagnosing HF was 13.71 ng/mL with a sensitivity of 77.0% and a specificity of 80.0%. To investigate the risk factors of HF patients, logistic stepwise regression analysis was performed after excluding the confounding factors between patients with worsening chronic HF and control group. As shown in Table 2, SFRP2 was positively associated with the odds of HF, whereas Hb, TC, and eGFR showed negative associations with the odds of HF. Finally, the results showed that higher SFRP2 (OR 1.05, 95% CI 1.02–1.08; *P* = 0.002) was independent predictor of HF after adjusting for Hb, TC, and eGFR.

**Table 2:** Logistic regression analysis of risk factors affecting HF.

Variables	Univariable analysis		Multivariable analysis	
	HR (95% CI)	*P* value	HR (95% CI)	*P* value
Hb	0.95 (0.93–0.97)	< 0.001	0.95 (0.93–0.98)	< 0.001
TC	0.34 (0.24–0.47)	< 0.001	0.36 (0.24–0.54)	< 0.001
eGFR	0.97 (0.96–0.98)	< 0.001	0.96 (0.94–0.98)	< 0.001
SFRP2	1.07 (1.03–1.10)	< 0.001	1.05 (1.02–1.08)	0.002

Hb, haemoglobin; TC, total cholesterol; eGFR, estimated glomerular filtration rate; NT-proBNP, N-terminal pro-B-type natriuretic peptide; SFRP2, secreted frizzled-related protein 2.

### Total endpoint events of HF during follow-up

In this study, two patients with worsening chronic HF were lost with follow-up information, the loss ratio was 1.24%. With a median follow-up of 450 (interquartile range 224 to 942) days, there were 72 (44.72%) endpoint events, comprising 25 (15.53%) all-cause mortality and 47 (29.19%)rehospitalizations owing to HF, acute myocardial infarction, and malignant arrhythmia. Furthermore, compared with patients without primary endpoint during follow-up, those with events were older, had higher proportion of male, had higher right atrial diameter, right ventricular diameter, NT-proBNP, and SFRP2 levels, were more likely to have a history of diabetes mellitus and ischemic heart disease (all *P* < 0.05). It was observed that there were no significant differences in other clinical characteristics between the two groups (Table 3).

A comparison of the characteristics of patients with/without the secondary endpoint was detailed in Table 4. Compared with patients without secondary endpoint, those with events were older, had higher diastolic blood pressure, heart rate and body mass index, had worse New York Heart Association and eGFR, had higher left ventricular diameter, NT-proBNP levels, were more likely to have a history of diabetes mellitus and hypertension (all *P* < 0.05). Similarly, the patients with the secondary endpoint were also found to have significantly higher SFRP2 levels. It was observed that there were no significant differences in other clinical characteristics between the two groups (Table 4).

**Table 3:** Baseline characteristics of the included HF patients stratified by primary endpoint.

	Primary endpoint (*n* = 25)	No primary endpoint (*n* = 136)	Ρ value
General characteristics			
Age (year)	80.04 ± 5.80	73.95 ± 8.53	0.001
Male (n,%)	20 (80.0)	76 (55.9)	0.024
Smoking (n,%)	13 (52.0)	48 (35.3)	0.114
SBP (mmHg)	130.00 (119.00,143.50)	132.00 (119.25,150.00)	0.698
DBP (mmHg)	70.00 (65.00,76.50)	74.00 (67.25,86.75)	0.057
Heart rate, bpm	86.00 (70.00,115.00)	85.00 (73.25,100.00)	0.821
BMI (kg/m^2^)	21.87 (19.66,25.09)	23.44 (21.63,25.40)	0.075
NYHA classification			0.187
II	1 (4.0)	17 (12.5)	
III	15 (60.0)	57 (41.9)	
IV	9 (36.0)	62 (45.6)	
Comorbidities			
DM (n,%)	13 (52.0)	43 (31.6)	0.049
Hypertension (n,%)	19 (76.0)	99 (72.8)	0.739
HF cause			
IHD (n,%)	16 (64.0)	52 (38.2)	0.017
Hypertensive heart disease (n,%)	8 (32.0)	61 (44.9)	0.233
DCM (n,%)	0 (0)	14 (10.3)	0.196
HCM (n,%)	2 (8.0)	7 (5.1)	0.923
Valvular heart disease (n,%)	7 (28.0)	24 (17.6)	0.228
Medications at discharge			
ACEIs (n,%)	0 (0)	5 (3.7)	0.729
ARBs (n,%)	5 (20.0)	54 (39.7)	0.060
Beta-blockers (n,%)	12 (48.0)	82 (60.3)	0.252
CCBs (n,%)	9 (36.0)	35 (25.7)	0.290
Aldosterone antagonist (n,%)	9 (36.0)	52 (38.2)	0.832
Diuretics (n,%)	12 (48.0)	74 (54.4)	0.555
Statins (n,%)	16 (64.0)	89 (65.4)	0.889
Antiplatelets (n,%)	5 (20.0)	39 (28.7)	0.371
Echocardiography			
LAD(cm)	4.56 ± 0.72	4.34 ± 0.67	0.150
LVD (cm)	4.89 (4.28,5.83)	4.82 (4.30,5.55)	0.657
RAD (cm)	4.36 ± 0.91	4.02 ± 0.74	0.045
RVD (cm)	3.73 ± 0.61	3.41 ± 0.58	0.015
EF (%)	54.70 (39.50,58.00)	55.00 (45.00,59.00)	0.419
LVEDD (mL)	97.00 (56.00,142.50)	83.00 (60.00,112.00)	0.481
Laboratory data			
WBC (×10^9^)	6.20 (5.35,7.30)	7.20 (5.40,8.60)	0.115
Neutrophil (×10^9^)	3.80 (3.20,4.95)	4.70 (3.53,6.15)	0.064
Hb (g/L)	117.64 ± 15.61	125.86 ± 24.56	0.109
lg(PLT) (×10^9^)	2.24 ± 0.13	2.28 ± 0.17	0.266
ALB (g/L)	37.64 ± 5.03	37.87 ± 4.80	0.829
lg(ALT) (U/L)	1.20 (1.02,1.35)	1.28 (1.11,1.46)	0.052
lg(AST) (U/L)	1.30 (1.18,1.47)	1.36 (1.26,1.48)	0.217
TG (mmol/L)	1.08 (0.72,1.24)	1.11 (0.87,1.46)	0.163
TC (mmol/L)	3.87 (3.21,4.67)	3.89 (3.16,5.01)	0.583
HDL-C (mmol/L)	1.17 (0.86,1.71)	1.08 (0.86,1.36)	0.434
LDL-C (mmol/L)	2.34 (1.88,2.98)	2.50 (1.73,3.31)	0.411
eGFR (mL/min/1.73 m^2^)	60.50 ± 30.21	70.23 ± 29.18	0.129
K^+^ (mmol/L)	4.13 ± 0.50	4.03 ± 0.47	0.331
Na^+^ (mmol/L)	142.0 (137.00,143.00)	141.00 (138.00,143.00)	0.558
lg (NT-proBNP) (pg/mL)	3.75 ± 0.46	3.46 ± 0.41	0.002
lg (SFRP2) (ng/mL)	1.51 (1.38,1.59)	1.31 (1.12,1.47)	0.002

Continuous variables are reported as mean (standard deviation) or median (interquartile range).Categorical variables were presented as number (percentages). P value < 0.05 is shown in bold type.SBP, systolic blood pressure; DBP, diastolic blood pressure; BMI, body mass index; NYHA, New York Heart Association; DM, diabetes mellitus; IHD, ischaemic heart disease; DCM, dilated cardiomyopathy; HCM, hypertrophic cardiomyopathy; WBC, white blood cell count; Hb, haemoglobin; PLT, platelet count; ALB, Albumin; ALT, alanine aminotransferase; AST, aspartate aminotransferase; TG, triglycerides; TC, total cholesterol; HDL-C, high-density lipoprotein cholesterol; LDL-C, low-density lipoprotein protein cholesterol; eGFR, estimated glomerular filtration rate; K+, potassium concentration; Na+, sodium concentration; ACEI, angiotensin converting enzyme inhibitor; ARB, angiotensin II receptor blocker; CCB, calcium channel blocker; LAD, Left atrial diameter; LVD, left ventricular diameter; RAD, right atrial diameter; RVD, right ventricular diameter; LVEF, left ventricular ejection fraction; LVEDD, left ventricular end-diastolic diameter; NT-proBNP, N-terminal pro-B-type natriuretic peptide; SFRP2, secreted frizzled-related protein 2.

**Table 4:** Baseline characteristics of the included HF patients stratified by secondary endpoint.

	Secondary endpoint (*n* = 72)	Nosecondary endpoint (*n* = 89)	Ρ value
General characteristics			
Age (year)	76.87 ± 7.19	73.25 ± 9.08	0.006
Male (n,%)	46 (63.0)	50 (56.8)	0.425
Smoking (n,%)	27 (37.0)	34 (38.6)	0.830
SBP (mmHg)	132.00 (120.50,147.00)	132.00 (118.25,150.75)	0.922
DBP (mmHg)	73.64 ± 11.26	78.13 ± 16.86	0.046
Heart rate, bpm	88.00 (76.00,111.00)	83.50 (71.00,96.00)	0.018
BMI (kg/m^2^)	88.00 (76.00,111.00)	83.50 (71.00,96.00)	0.018
NYHA classification			0.010
II	2 (2.8)	16 (18.0)	
III	35 (48.6)	37 (41.6)	
IV	35 (48.6)	36 (40.4)	
Comorbidities			
DM (n,%)	36 (49.3)	20 (22.7)	< 0.001
Hypertension (n,%)	61 (83.6)	57 (64.8)	0.007
HF cause			
IHD (n,%)	35 (47.9)	33 (37.5)	0.182
Hypertensive heart disease (n,%)	36 (49.3)	33 (37.5)	0.132
DCM (n,%)	6 (8.2)	8 (9.1)	0.845
HCM (n,%)	3 (4.1)	6 (6.8)	0.689
Valvular heart disease (n,%)	14 (19.2)	17 (19.3)	0.982
Medications at discharge			
ACEIs (n,%)	1 (1.4)	4 (4.5)	0.484
ARBs (n,%)	25 (34.2)	34 (38.6)	0.416
Beta-blockers (n,%)	45 (61.6)	49 (55.7)	0.888
CCBs (n,%)	26 (35.6)	18 (20.5)	0.302
Aldosterone antagonist (n,%)	22 (30.1)	39 (44.3)	0.086
Diuretics (n,%)	36 (49.3)	50 (56.8)	0.286
Statins (n,%)	50 (68.5)	55 (62.5)	0.886
Antiplatelets (n,%)	14 (19.2)	30 (34.1)	0.065
Echocardiography			
LAD (cm)	4.50 ± 0.66	4.28 ± 0.69	0.045
LVD (cm)	4.84 (4.29,5.63)	4.82 (4.32,5.50)	0.705
RAD (cm)	4.16 ± 0.81	4.00 ± 0.74	0.185
RVD (cm)	3.50 (3.09,3.84)	3.37 (3.02,3.78)	0.114
EF (%)	54.00 (40.50,59.00)	55.00 (47.25,59.00)	0.336
LVEDD (mL)	87.00 (60.50,124.00)	79.50 (60.00,108.00)	0.506
Laboratory data			
WBC (×10^9^)	7.18 ± 2.24	7.03 ± 2.16	0.679
Neutrophil (×10^9^)	4.60 (3.40,5.75)	4.60 (3.50,5.98)	0.771
Hb (g/L)	121.84 ± 20.55	126.86 ± 25.66	0.178
lg(PLT) (×10^9^)	2.28 ± 0.16	2.28 ± 0.17	0.986
ALB (g/L)	38.23 ± 4.68	37.50 ± 4.94	0.338
lg (ALT) (U/L)	1.26 ± 0.29	1.29 ± 0.29	0.537
lg (AST) (U/L)	1.36 ± 0.20	1.38 ± 0.23	0.646
TG (mmol/L)	1.16 (0.81,1.53)	1.07 (0.81,1.43)	0.126
TC (mmol/L)	3.80 (3.02,4.70)	4.07 (3.16,4.92)	0.187
HDL-C (mmol/L)	1.09 (0.86,1.40)	1.08 (0.86,1.38)	0.879
LDL-C (mmol/L)	1.09 (0.86,1.40)	2.58 (1.90,3.29)	0.171
eGFR (mL/min/1.73 m^2^)	62.07 ± 26.93	74.24 ± 30.47	0.009
K^+^ (mmol/L)	4.04 ± 0.48	4.05 ± 0.47	0.901
Na^+^ (mmol/L)	141.00 (138.00,143.00)	141.00 (138.00,143.00)	0.623
Lg (NT-proBNP) (pg/mL)	3.62 ± 0.40	3.41 ± 0.42	0.001
Lg (SFRP2) (ng/mL)	1.44 (1.24,1.57)	1.28 (1.08,1.43)	< 0.001

Continuous variables are reported as mean (standard deviation) or median (interquartile range).Categorical variables were presented as number (percentages). P value < 0.05 is shown in bold type.SBP, systolic blood pressure; DBP, diastolic blood pressure; BMI, body mass index; NYHA, New York Heart Association; DM, diabetes mellitus; IHD, ischaemic heart disease; DCM, dilated cardiomyopathy; HCM, hypertrophic cardiomyopathy; WBC, white blood cell count; Hb, haemoglobin; PLT, platelet count; ALB, Albumin; ALT, alanine aminotransferase; AST, aspartate aminotransferase; TG, triglycerides; TC, total cholesterol; HDL-C, high-density lipoprotein cholesterol; LDL-C, low-density lipoprotein protein cholesterol; eGFR, estimated glomerular filtration rate; K+, potassium concentration; Na+, sodium concentration; ACEI, angiotensin converting enzyme inhibitor; ARB, angiotensin II receptor blocker; CCB, calcium channel blocker; LAD, Left atrial diameter; LVD, left ventricular diameter; RAD, right atrial diameter; RVD, right ventricular diameter; LVEF, left ventricular ejection fraction; LVEDD, left ventricular end-diastolic diameter; NT-proBNP, N-terminal pro-B-type natriuretic peptide; SFRP2, secreted frizzled-related protein 2.

### Prognostic value of SFRP2 for the prediction of endpoint events in elderly patient with worsening chronic HF

**Fig. 2:**
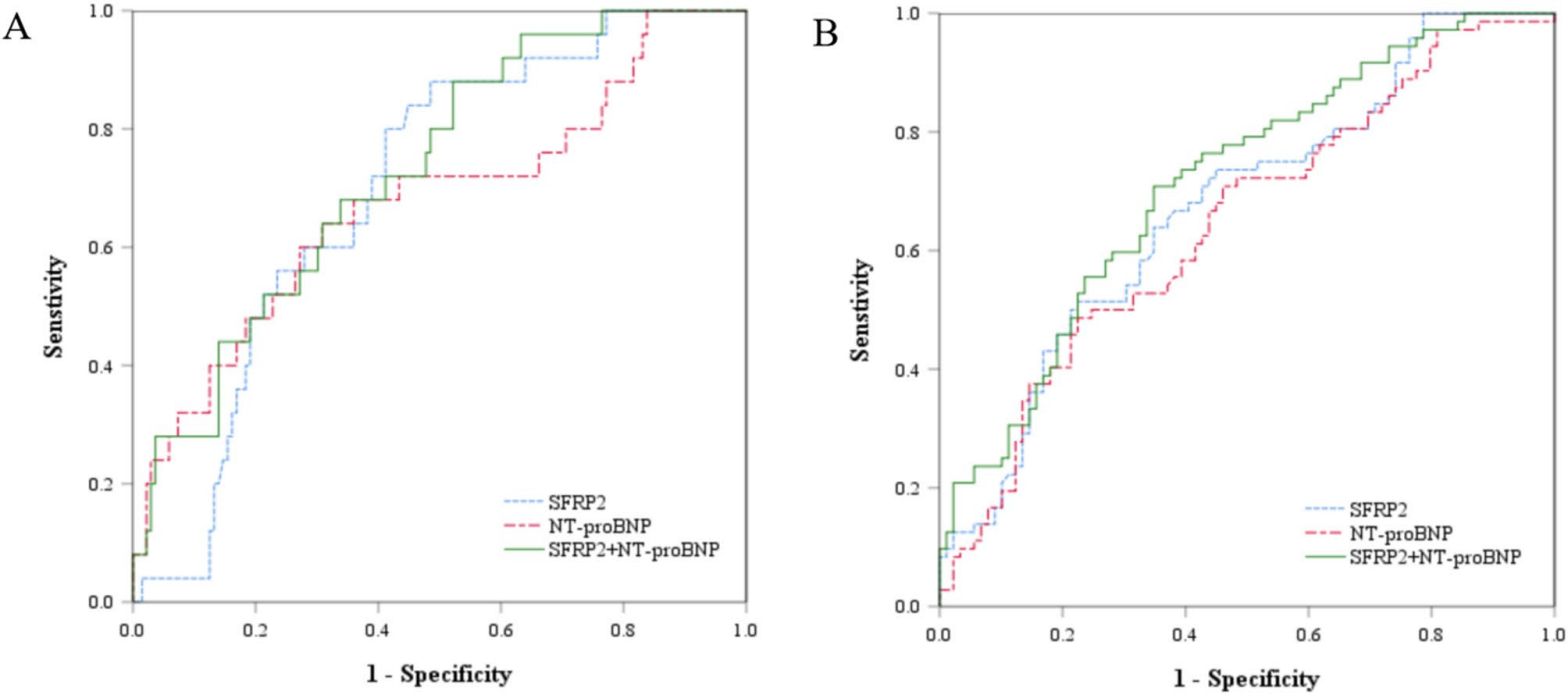
Receiver operating characteristic (ROC) curve analyses of SFRP2, NT-proBNP, and SFRP2 plus NT-proBNP for the prediction of (**A**) primary endpoint (**B**) secondary endpoint. SFRP2, secreted frizzled-related protein 2; NT-proBNP, N-terminal pro-B-type natriuretic peptide.

In this study, the abilities of SFRP2 and NT-proBNP to identify patients at higher risk of adverse events were compared. Based on the ROC analysis, we determined that the optimal cut-off value of SFRP2 level was 20.85 ng/mL, with an AUC of 0.700 (95% CI 0.605–0.795;*P* = 0.002) for predicting primary endpoint and the optimal SFRP2 value was 23.63 ng/mL, with an AUC of 0.674 (95% CI 0.591–0.757;*P* < 0.002) in secondary endpoint. Described by the AUC, the discriminatory power of NT-proBNP to distinguish patients with and without primary endpoint was 0.676 (95% CI 0.552–0.801;*P* = 0.005). ROC analysis also showed that level of NT-proBNP (AUC = 0.644, 95% CI 0.559–0.730;*P* = 0.002) had a significant predictive role on the secondary endpoint. Similarly, the AUC value of SFRP2 in combination with NT-proBNP for predicting the primary endpoint and secondary endpoint were 0.730 (95% CI 0.631–0.828;*P* < 0.001) and 0.713 (95% CI = 0.635–0.792;*P* < 0.001), respectively. We revealed no significant differences among the three parameters in ROC (Fig. 2, all *P* for pairwise comparisons of AUCs > 0.05).

**Fig. 3:**
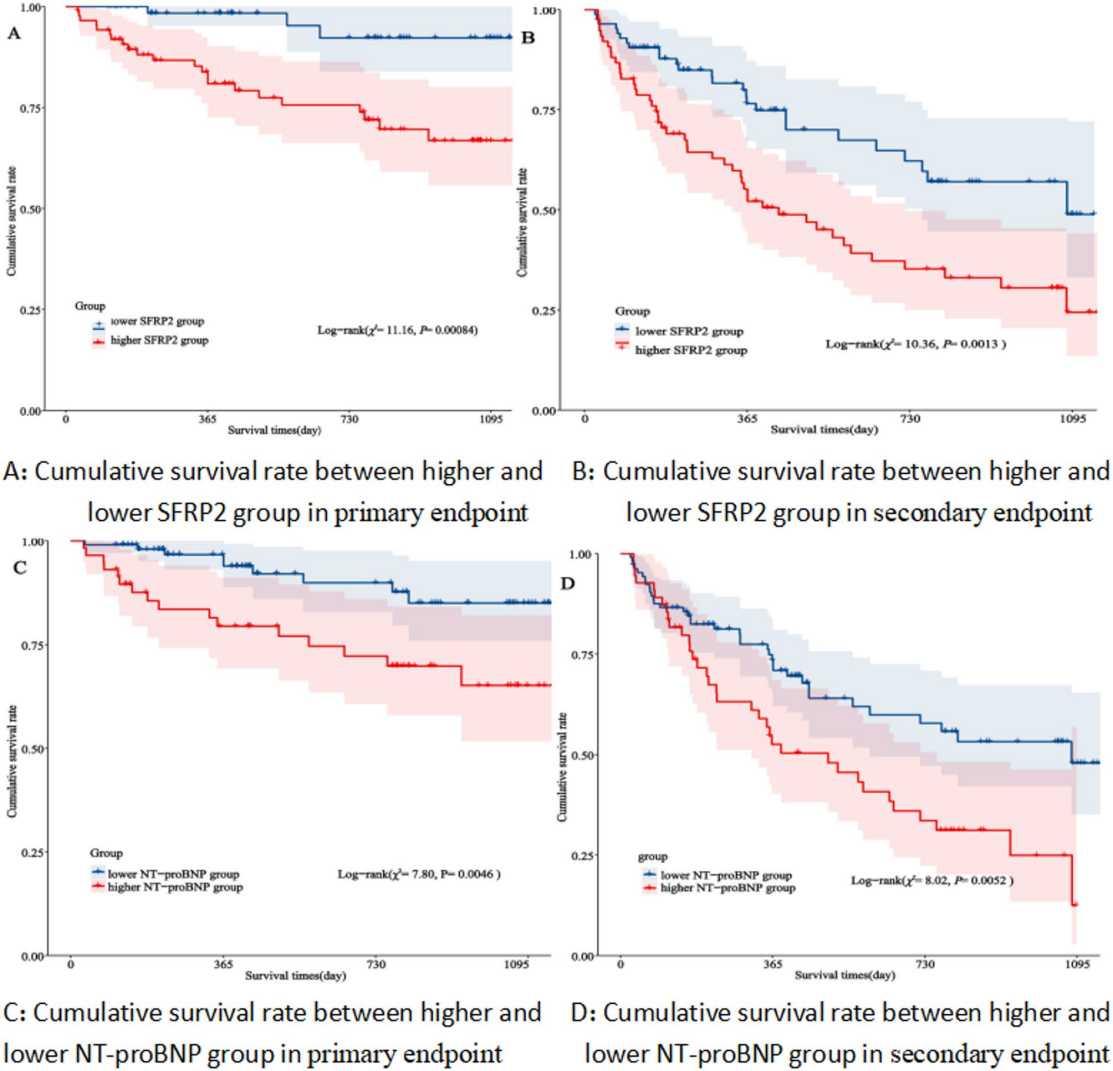
Kaplan-Meier analysis of (**A**) primary endpoint between the patients with SFRP2 < 20.85 ng/mL and SFRP2 ≥ 20.85 ng/mL(*χ*^*2*^= 11.16, *P*= 0.00084 ), and (**B**) secondary endpoint between the patients with SFRP2 < 23.63 ng/mL and SFRP2 ≥ 23.63 ng/mL(*χ*^*2*^= 10.36, *P*= 0.0013 ), (**C**) primary endpoint between the patients with NT-proBNP < 4522.5 pg/mL and NT-proBNP ≥ 4522.5 pg/mL(*χ*^*2*^= 7.80, *P*= 0.0046 ), and (**D**) secondary endpoint between the patients with NT-proBNP < 4673.50 pg/mL and NT-proBNP ≥ 4673.50 pg/mL(*χ*^*2*^= 8.02, *P*= 0.0052 ). SFRP2, secreted frizzled-related protein 2; NT-proBNP, N-terminal pro-B-type natriuretic peptide.

We compared the baseline of SFRP2 and NT-proBNP to find their relationship with the risk of adverse outcomes. All patients were divided into two groups according to the optimal cut-off value of SFRP2 and NT-proBNP through the ROC curve analysis. In the Kaplan-Meier analysis, patients in the high SFRP2 and NT-proBNP level groups had a significantly greater risk of primary endpoint and secondary endpoint and a lower survival rate than those in low SFRP2 and NT-proBNP level groups (log-rank test: *P* = 0.00084, 0.0013, 0.0046, and.0.0052, respectively, Fig. 3A–D).

### Prognostic advantage of SFRP2 and the risk factors of endpoint evens in elderly patients with heart failure

Using the univariable Cox regression, we identified parameters (age, male, smoking, ischaemic heart disease, right ventricular diameter, lg(NT-proBNP), and SFRP2 that were significantly associated with primary endpoint. These parameters were used as inputs for the multivariable COX stepwise regression analysis. In multivariable Cox regression models, age (hazard ratio (HR) 1.087, 95% CI 1.026–1.152;*P* = 0.005), history of smoking (HR 2.334, 95% CI 1.059–5.147;*P* = 0.036), higher lg (NT-proBNP) (HR 4.783, 95% CI 1.682–13.604;*P* = 0.003), and SFRP2 (HR 2.334, 95% CI 1.059–5.147;*P* = 0.036) were independent risk factors for the primary endpoint (Table 5).

As shown in Table 6, eGFR was negatively associated with the secondary endpoint, whereas hypertension, diabetes mellitus, use of calcium channel blocker, treatment with statins, lg(NT-proBNP), and SFRP2 showed positive associations with the secondary endpoint. Moreover, the final multivariable Cox proportional hazards regression model was created using a forward stepwise algorithm that included presence of diabetes mellitus (HR 1.897, 95% CI 1.180–3.048;*P* = 0.008), treatment with statins (HR 1.851, 95% CI 1.113–3.078; *P* = 0.018), higher lg(NT-proBNP) (HR 2.041, 95% CI 1.189–3.502;*P* = 0.010), and SFRP2 (HR 2.326, 95% CI 1.426–3.794;*P* = 0.001) levels.

**Table 5:** The association between risk factors and primary endpoint in HF patients.

Variables	Univariable analysis		Multivariable analysis	
	HR (95% CI)	*P* value	HR (95% CI)	*P* value
Age	1.084 (1.028–1.144)	0.003	1.087 (1.026–1.152)	0.005
Sex	3.124 (1.205–8.574)	0.020		
Smoking	2.318 (1.005–5.095)	0.036	2.334 (1.059–5.147)	0.036
IHD	2.884 (1.272–6.540)	0.011		
RVD	2.204 (1.199–4.049)	0.011		
lg(NT-proBNP)	4.328 (1.597–11.726)	0.004	4.783 (1.682–13.604)	0.003
SFRP2 ≥ 20.85 ng/mL	6.068 (1.815–20.288)	0.003	2.334 (1.059–5.147)	0.036

IHD, ischaemic heart disease; RVD, right ventricular diameter; NT-proBNP, N-terminal pro-B-type natriuretic peptide; SFRP2, secreted frizzled-related protein 2.

**Table 6:** The association between risk factors and secondary endpoint in HF patients.

Variables	Univariable analysis		Multivariable analysis	
	HR (95% CI)	*P* value	HR (95% CI)	*P* value
Hypertension	2.106 (1.131–3.921)	0.019		
DM	2.232 (1.403–3.551)	0.001	1.897 (1.180–3.048)	0.008
CCB	1.687 (1.035–2.750)	0.036		
Statins	1.635 (0.990–2.701)	0.055	1.851 (1.113–3.078)	0.018
eGFR	0.990 (0.982–0.998)	0.017		
lg(NT-proBNP)	1.970 (1.132–3.429)	0.016	2.041 (1.189–3.502)	0.010
SFRP2 ≥ 23.63 ng/mL	2.161 (1.335–3.497)	0.002	2.326 (1.426–3.794)	0.001

DM, diabetes mellitus; CCB, calcium channel blocker; eGFR, estimated glomerular filtration rate; NT-proBNP, N-terminal pro-B-type natriuretic peptide; SFRP2, secreted frizzled-related protein 2.

## Discussion

In the current study, SFRP2 increased significantly in elderly patients with worsening chronic HF compared with control subjects. Importantly, it was noteworthy that the elderly worsening chronic HF patients in the base high-level SFRP2 had a significantly greater risk of poor outcomes and a lower survival rate.Of particular significance, the SFRP2 value at admission was a powerful and independent predictor of primary endpoint and secondary endpoint in elderly patients with worsening chronic HF, even after adjusting for other confounding factors, including plasma NT-proBNP levels. To the best of our knowledge, this study is the first to demonstrate that an increased level of SFRP2 was associated with worse prognosis of elderly patients with worsening chronic HF. On the other hand, our study was supported by another prospective cohort study, which provided the message that SFRP2 was a marker of myocardial fibrosis detected by cardiovascular magnetic resonance in HF patients^12^. Although SFRP2 had statistical significance in predicting the prognosis of heart failure patients, its hazard ratio (HR 2.334) was not large, and its role in predicting the prognosis of heart failure was limited. It had not been confirmed by other peer studies or randomized controlled trials, and cannot completely replace the traditional indicator NT-proBNP in predicting the prognosis of heart failure. However, the addition of SFRP2 may enhance the prognostic value of NT-proBNP.

Myocardial fibrosis played an important role in the pathogenesis of HF and represented a potential therapeutic target for HF^17,18^. Several potential mechanisms that involved the interaction between SFRP2 and myocardial fibrosis supported our observations. The level of fibrosis in the infarcted heart was markedly reduced in SFRP2-null mice, which is accompanied by significantly improved cardiac function^19^. Similarly, the work of Lin et al.. reported that SFRP2 activated adult mouse cardiac fibroblasts via the Wnt/β-catenin signaling pathways^4^. Meanwhile, an antibody-based SFRP2 blockade strategy could also counteract the fibrogenic pathway and repair cardiac injury in the failing hamster heart^20^. It has also been proposed that SFRP2 promoted cardiac fibrocalcification through coordinate activation of tolloid-like metalloproteinases and tissue nonspecific alkaline phosphatase^21^. In contrast, Cao et al.. revealed that the decreased circulating SFRP2 was a risk factor for HF in patients with cardiovascular diseases^13^. Other studies had shown that over expression of SFRP2 could inhibit myocyte fibrosis and induce angiogenesis^22–24^. This hypothesis was supported by findings that the effect of SFRP2 on myocardial fibrosis existed bidirectional relation and was concentration dependent^25^. This suggested a context-dependent role of SFRP2: protective in acute ischemic conditions but profibrotic in non-ischemic settings.

On the basis of past research showed that NT-proBNP played an important role in the diagnosis and prognosis of HF. Accordingly, our study observed that the plasma NT-proBNP levels were significantly higher in patients with endpoint events than those without endpoint events, which was in accordance with the PARADIGM-HF study^26^. However, NT-proBNP was susceptible to age, renal dysfunction, obesity^27^. Therefore, discovering and developing new prognostic indicators for heart failure that were not affected by age, kidney function, obesity, etc. has become a new direction in the field of heart failure biomarker research. Our study further demonstrated that the combination of SFRP2 and NT-proBNP was a stronger predictive for endpoint events in elderly patients with worsening chronic HF. SFRP2 had a higher risk relativation in the secondary endpoint in HF patients compared to NT-proBNP. Combining SFRP2 with NT-proBNP improved risk stratification (AUC increased from 0.0.674 to 0.0.713, *P* < 0.01), outperforming conventional biomarkers in identifying high-risk patients with preserved NT-proBNP levels. SFRP2 regulated myocardial fibrosis and inflammation through the Wnt/β-catenin pathway, which was distincted from the ventricular wall stress pathway reflected by NT-proBNP. This unique mechanism suggested SFRP2 may capture complementary prognostic information. While Barcelona-HF and MAGGIC integrated clinical variables, they lacked dynamic biomarker data. SFRP2’s role in myocardial remodeling offered a pathophysiological link to outcomes that scores may miss. Thus, SFRP2 may provide more additional information beyond NT-proBNP in the prognostic assessment of HF.

Strong evidence already existed that the risk of all-cause mortality was higher in cardiovascular disease patients who were smokers and nominally so also in older patients^28^.Obviously, similar results were observed in elderly patients with worsening chronic HF in our study. This was unsurprising because age and smoking were important risk factors correlated with HF.

Diabetes mellitus is an increasingly common comorbidity in HF and worsened the prognosis of HF^29^. Pocock et al.. found that the presence of DM in HF patients was independently associated with higher rates of rehospitalization^30^.It was noteworthy that glycemia did not significantly predict 1year all-cause mortality in the cohort of more than 50,000 elderly patients with HF^31^. Likewise, another study supported the idea that DM had no impact on short-term mortality in HF patients, but the rehospitalization rate of HF patients was higher in those with DM^32^. Our study shared similarities with previous observations that HF patients with DM had an increased risk of rehospitalization compared to patients without DM after adjustment for potential confounding, whereas there were no significant differences in all-cause mortality by DM.

The efficacy of statins on HF remained unclear.Two large clinical trials reported that patients with established HF had not been shown to benefit from statins^33,34^. Recently, a review revealed that statins didn’t reduce the risk of cardiovascular death in patients with a history of HF^35^. However, another meta-analysis suggested that statins might improve cardiovascular outcomes in HF patients^36^.In our present study, there was significant increment from statins therapy for all-cause death as well as rehospitalization for HF, acute myocardial infarction, and malignant arrhythmias.

Summary, SFRP2, NT-proBNP, smoking, and age at admission provided independent and complementary prognostic information in elder patients with HF^37^.

Some potential limitations in our study should be acknowledged. First, as a single-center prospective study, the current research may be susceptible to selection and confirmation bias. Second, the number of events were relatively small, thereby limiting the generalizability of the results, limiting the subanalysis on age, gender, comorbidities and limiting the subanalysis on normal or near-normal NT-proBNP patients. A post-hoc power analysis using G*Power (α = 0.05, effect size = 0.3) indicated a power of 88%, suggesting adequate sensitivity for primary endpoints(The study had 161 patients and 25 events, the recalculated requirement is 142 patients and 22 events). Third, the level of SFRP2 was only measured when they were admitted to the hospital, not during follow-up, which affected the judgment of the long-term value of this index. Fourth, due to medical insurance reimbursement, it was difficult to obtain SGLT2 Inhibitors, ARNI and vericiguat in our region. Its absence may underestimate potential prognostic interactions with SFRP2, especially given shared pathways in myocardial remodeling. Fifth, due to the exclusion of individuals with severe hepatic or renal dysfunction and acute coronary syndrome from the study population, the generalizability of the conclusions of this study to similar populations was limited. Lastly, more clinical and experimental studies were warranted to further explore the relationship between SFRP2 and worse clinical outcomes in elderly patients with worsening chronic HF.

## Data Availability

The data used to support the findings of this study are not publicly available because they are part of a larger data set which is being reported separately but are available from the corresponding authors upon a reasonable request.
